# Editorial: COVID-19 pandemic: A curve ball for athletes

**DOI:** 10.3389/fspor.2022.1015938

**Published:** 2022-09-08

**Authors:** Roy T. H. Cheung, Shirley P. C. Ngai, Heiner Baur

**Affiliations:** ^1^School of Health Sciences, Western Sydney University, Penrith, NSW, Australia; ^2^Department of Rehabilitation Sciences, The Hong Kong Polytechnic University, Kowloon, Hong Kong SAR, China; ^3^School of Health Professions, Bern University of Applied Sciences, Bern, Switzerland

**Keywords:** sportsmen, training, lifestyle, COVID-19, lockdown 2020

More than 30 months since the discovery of the novel respiratory coronavirus in 2019, COVID-19 related public health orders and restrictions remain active in many countries on the globe in late 2022. These measures, such as city lockdown, border closure, travel restrictions, social distancing practice, and mandatory use of facemasks, affect all segments of the population. During the pandemic, we have witnessed the most significant disruption to the worldwide sports calendar since the World War II. From a global perspective, many international sports events, such as The Olympic Games Tokyo 2020, Summer World University Games, and the World Games have been postponed; and more than 150 international sports events involving both professional and recreational sportsmen have been canceled. In this series, we cover original articles examining the effect of COVID-19 on the training routine and performance in five types of athletes, including distance runners (Chan et al.), soccer/football players (Wagemans et al.; Keemss et al.), bodybuilders (Imboden et al.; Iff et al.), volleyball players (Morath et al.), and paralympic athletes (Busch et al.). We are aware that this field of research is highly dynamic with new data available almost on a daily base. Hence, we aim to be more inclusive in this Research Topic and involve a wider scope of research questions and different methodological approaches, which allow us a better coverage of this emerging and evolving Research Topic.

These findings provide important information for athletes, coaches, physical trainers, and healthcare team members to identify potential health issues that may be related to the pandemic, plan specifically how we can minimize the negative influence, as well as design contingency training plan for postponed tournaments.

Although COVID-19 attacks our respiratory system and potentially causes a decline in physical condition, we observed adverse findings from the studies in this Research Topic in terms of physical training. Iff et al. and Keemss et al. reported a pandemic related negative impact on the physical performance in body builders and youth soccer players, respectively. In contrast, Chan et al. and Wagemans et al. did not find any substantial differences in terms of physical function or training intensity in professional soccer players and recreational distance runners. Interestingly, it seems that COVID-19 and its related public health restrictions result in a greater influence on people's mental than physical health. For example, Imboden et al., Busch et al., and Iff et al. reported that athletes exhibited poorer mood during the pandemic and this psychological impact may indeed lead to a change in living habits, such as increase alcoholic and cannabis intake. From a global perspective, this Research Topic also includes an investigation of COVID transmission within volleyball games. Morath et al. conducted contact tracing in a professional volleyball team in Germany. They found that players who strictly adhere to the recommended hygiene guidelines and regulations during both training and matches are of lower risk contracting the virus, but coaches and players are advised to avoid non-essential interpersonal contacts outside the training hours to prevent the spread of infection.

Although the guest editors would love to see more views, downloads, and citations of papers included in this series, we sincerely hope that athletes, coaches, and concerning healthcare professionals do not require the information presented in this Research Topic due to another wave of pandemic and disruption. May COVID-19 will be soon behind us and becomes a historical terminology in near future.

## Author contributions

RC had the first draft of this manuscript. HB and SN commented and revised the draft. All authors approved the final version of the manuscript.

## Conflict of interest

The authors declare that the research was conducted in the absence of any commercial or financial relationships that could be construed as a potential conflict of interest.

## Publisher's note

All claims expressed in this article are solely those of the authors and do not necessarily represent those of their affiliated organizations, or those of the publisher, the editors and the reviewers. Any product that may be evaluated in this article, or claim that may be made by its manufacturer, is not guaranteed or endorsed by the publisher.

